# Evaluation of a Diabetes Remote Monitoring Program Facilitated by Connected Glucose Meters for Patients With Poorly Controlled Type 2 Diabetes: Randomized Crossover Trial

**DOI:** 10.2196/25574

**Published:** 2021-03-11

**Authors:** Daniel J Amante, David M Harlan, Stephenie C Lemon, David D McManus, Oladapo O Olaitan, Sherry L Pagoto, Ben S Gerber, Michael J Thompson

**Affiliations:** 1 Department of Population and Quantitative Health Sciences University of Massachusetts Medical School Worcester, MA United States; 2 Division of Diabetes Department of Medicine University of Massachusetts Medical School Worcester, MA United States; 3 Division of Cardiovascular Medicine Department of Medicine University of Massachusetts Medical School Worcester, MA United States; 4 Department of Allied Health Sciences Institute for Collaborations on Health, Interventions, and Policy University of Connecticut Storrs, CT United States; 5 Department of Medicine University of Illinois at Chicago Chicago, IL United States

**Keywords:** self-monitoring, blood glucose, telemedicine, type 2 diabetes, diabetes, remote monitoring, support, adult

## Abstract

**Background:**

Patients with poorly controlled type 2 diabetes (T2D) experience increased morbidity, increased mortality, and higher cost of care. Self-monitoring of blood glucose (SMBG) is a critical component of diabetes self-management with established diabetes outcome benefits. Technological advancements in blood glucose meters, including cellular-connected devices that automatically upload SMBG data to secure cloud-based databases, allow for improved sharing and monitoring of SMBG data. Real-time monitoring of SMBG data presents opportunities to provide timely support to patients that is responsive to abnormal SMBG recordings. Such diabetes remote monitoring programs can provide patients with poorly controlled T2D additional support needed to improve critical outcomes.

**Objective:**

To evaluate 6 months of a diabetes remote monitoring program facilitated by cellular-connected glucose meter, access to a diabetes coach, and support responsive to abnormal blood glucose recordings greater than 400 mg/dL or below 50 mg/dL in adults with poorly controlled T2D.

**Methods:**

Patients (N=119) receiving care at a diabetes center of excellence participated in a two-arm, 12-month randomized crossover study. The intervention included a cellular-connected glucose meter and phone-based diabetes coaching provided by Livongo Health. The coach answered questions, assisted in goal setting, and provided support in response to abnormal glucose levels. One group received the intervention for 6 months before returning to usual care (IV/UC). The other group received usual care before enrolling in the intervention (UC/IV) for 6 months. Change in hemoglobin A_1c_ (HbA_1c_) was the primary outcome, and change in treatment satisfaction was the secondary outcome.

**Results:**

Improvements in mean HbA_1c_ were seen in both groups during the first 6 months (IV/UC −1.1%, SD 1.5 vs UC/IV −0.8%, SD 1.5; *P*<.001). After crossover, there was no significant change in HbA_1c_ in IV/UC (mean HbA_1c_ change +0.2, SD 1.7, *P*=.41); however, those in UC/IV showed further improvement (mean HbA_1c_ change −0.4%, SD 1.0, *P*=.008). A mixed-effects model showed no significant treatment effect (IV vs UC) over 12 months (*P*=.06). However, participants with higher baseline HbA_1c_ and those in the first time period experienced greater improvements in HbA_1c_. Both groups reported similar improvements in treatment satisfaction throughout the study.

**Conclusions:**

Patients enrolled in the diabetes remote monitoring program intervention experienced improvements in HbA_1c_ and treatment satisfaction similar to usual care at a specialty diabetes center. Future studies on diabetes remote monitoring programs should incorporate scheduled coaching components and involve family members and caregivers.

**Trial Registration:**

ClinicalTrials.gov NCT03124043; https://clinicaltrials.gov/ct2/show/NCT03124043

## Introduction

Poorly controlled diabetes, as indicated by elevated hemoglobin A_1c_ (HbA_1c_), is associated with higher morbidity and mortality [1], greater cost of treatment [2], and poorer adherence to recommended self-management behaviors [3]. To improve HbA_1c_, diabetes self-management support needs to be accessible, responsive to varying patient health status, and effective in improving self-management skills, knowledge, and engagement. This is especially important for patients who struggle with self-management or face barriers to accessing traditional in-person services due to social determinants of health [4]. Integrated health care systems and payers, including commercial health plans, are particularly interested in innovative approaches to self-management support that address diabetes quality measures while reducing the overall cost of care [5]. Consequently, various commercial products have been developed to improve diabetes self-management, improve the experience of care, and reduce overall costs.

Electronic remote patient monitoring is a common strategy for many diabetes self-management applications available. This generally involves the transmission of self-monitored blood glucose readings to health care professionals and teams for evaluation and feedback [6]. Such real-time provider access to patient monitoring data presents an opportunity for care teams to deliver timely, tailored support without in-person contact. However, additional research targeting provider behavior with consideration of reimbursement for time and effort is needed to successfully integrate remote monitoring into routine care [7]. A recent meta-analysis of 4 systematic reviews of randomized controlled trials evaluating phone- and internet-based monitoring found improvement in HbA_1c_ levels of −0.55% (95% CI −0.73 to −0.36) compared with usual care, though with statistical heterogeneity [6]. Notably, only 14 of 25 randomized trials reported significant improvement over usual care, with variability in what usual care support entails, as well as study quality. Potentially, positive findings may represent substandard care in comparison groups and may reflect the lack of resources required to ensure adequate evaluation and feedback is given to patients.

The Livongo for Diabetes Program is commercially available for purchase for individual use or can be implemented through a health organization or insurer. The program highlights the integration of Certified Diabetes Educators (CDEs), also referred to as Certified Diabetes Care and Education Specialists, who can provide real-time feedback on glucose monitoring data, including immediate responses to abnormal glucose excursions. One prior observational study of over 4500 individuals with diabetes using the Livongo for Diabetes Program found a decrease in glucose levels outside of a 70-180 mg/dL range [8]. However, the study did not include a comparison group to establish efficacy, and HbA_1c_ was not assessed to understand if there was less hypoglycemia, less hyperglycemia, or both.

The present study was a randomized controlled crossover trial testing the efficacy of 6 months of participation in the Livongo for Diabetes Program in patients with poorly controlled type 2 diabetes. The primary outcome of the trial was change in HbA_1c_, with a secondary outcome of change in diabetes treatment satisfaction. In this study, we hypothesized that patients would experience greater improvements in HbA_1c_ and treatment satisfaction when enrolled in the intervention program compared to usual care. Additionally, we explored engagement with the program, including monitor use and receipt of CDE support.

## Methods

### Setting and Recruitment

Participants with type 2 diabetes were recruited at the University of Massachusetts Medical Center Diabetes Center of Excellence (DCOE) from April 1 to July 9, 2015. All patients at the DCOE have both a primary care provider and a DCOE specialist provider. Inclusion criteria included the ability to speak English, a diagnosis of type 2 diabetes, and two consecutive HbA_1c_ recordings greater than 8.0% in the previous 12 months, indicating poor glycemic control. Subjects were excluded if they were cognitively impaired (as designated by their provider), pregnant, or a prisoner. All human subjects research was reviewed and approved by the University of Massachusetts Medical School Institutional Review Board.

Research staff screened medical records of patients scheduled for routine appointments to identify those meeting the HbA_1c_ criterion. The staff approached potentially eligible patients in the clinical environment and privately screened for eligibility if patients expressed interest. Patients were informed that they would be given access to the Livongo for Diabetes Program for a total of 6 months, either immediately or after a 6-month waiting period, randomly determined. Interested and eligible participants signed consent forms. Of 195 eligible subjects approached for recruitment, 123 (63.1%) expressed interest in participating, and 120 (61.5%) completed the informed consent process and were randomized to treatment groups. One subject failed to complete the baseline survey and was lost to follow-up prior to enrollment in the intervention.

### Intervention

The intervention included free enrollment in the Livongo for Diabetes Program [9], the Livongo In Touch connected glucose meter, and a 6-month supply of testing supplies. The Livongo for Diabetes Program is accredited by the American Association of Diabetes Educators (AADE) Diabetes Education Accreditation Program and includes access to both scheduled and in-the-moment CDE support via phone call or SMS text messaging. At the time of the study, the Livongo for Diabetes program was not available as a direct-to-consumer product but was available to employees of several large companies.

The In Touch connected glucose meter is cellular-enabled, allowing for automatic uploading of self-monitoring of blood glucose (SMBG) recordings to a secure patient portal. Patients were instructed to use the meter to test their blood glucose as frequently as previously instructed by their providers. After patients use the meter to test their glucose, the SMBG recording is uploaded to the Livongo Smart Cloud. In this study, Livongo transferred all SMBG data to the DCOE electronic health record (EHR) system daily. The first time an uploaded blood glucose recording was above 250 mg/dL and anytime it was above 400 mg/dL or below 50 mg/dL, the Livongo Smart Cloud would notify the Livongo Care Team to perform outreach to the patient.

**Figure 1:**
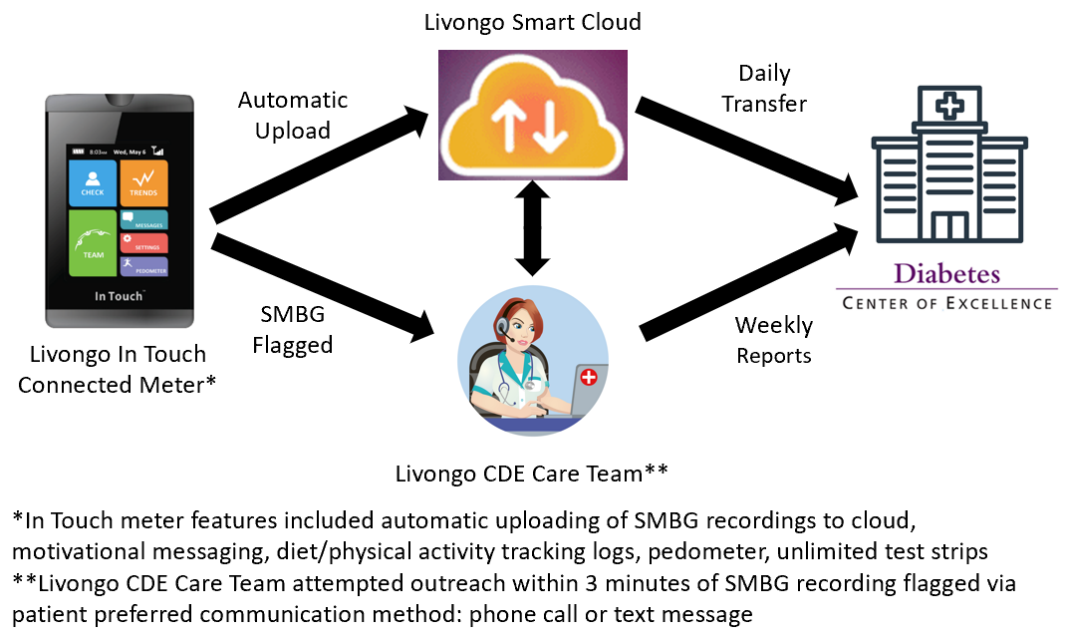
Intervention components and flow of patient data. CDE: Certified Diabetes Educator; SMBG: self-monitoring of blood glucose.

The Livongo Care Team of CDEs would contact participants by their preferred communication method (either phone call or text message) within 3 minutes of receiving an abnormal SMBG notification from the Smart Cloud. When contact was made, they would assess if the patient needed immediate medical attention, troubleshoot reasons for the flagged SMBG recording, and provide resources to improve self-management of diabetes. If a participant needed immediate medical attention, the CDE would direct them to call 911. If the intervention CDE believed a participant was in need of additional support from their DCOE care team, the CDE would contact the DCOE directly to request follow-up with the patient. Documentation of all encounters between intervention CDEs and participants was sent to the DCOE weekly to be entered into the EHR (Figure 1 for intervention components and flow of data). While the SMBG and CDE encounter data were available to the DCOE providers, the study did not target DCOE provider behavior (eg, by encouraging the providers to review or use the intervention data available in the EHR).

Intervention participants were encouraged at enrollment and during each CDE outreach to schedule follow-up coaching sessions with the CDEs. Coaching sessions covered the AADE’s 7 self-care behaviors: healthy eating, being active, glucose monitoring, taking medication, problem solving, reducing risks, and health coping [10]. While intervention CDEs did not give participants medical direction or make changes to their care plans, they answered diabetes-specific questions on topics such as nutrition and lifestyle changes and contacted the DCOE if they believed the participant would benefit from additional medical intervention.

Text-based messages sent to the participants through the meter after each test were based on the AADE National Standards for Diabetes Self-Management Education curriculum and included feedback and diabetes self-management tips. Other features of the meter included tagging SMBG recordings with contextual information (before meal, after meal, neither, and how they were feeling at the time of testing), an electronic logbook, and a built-in physical activity tracker. The meter also allowed participants to share SMBG data with their care providers or family via text message, email, or fax. While Livongo now offers a mobile phone app to accompany the In Touch meter, this app was not available at the time of the study.

### Usual Care

Participants in the usual care group continued to receive specialty care from DCOE and primary care providers. This included the recommended quarterly appointments with their DCOE care team and regular access to their providers through phone calls or secure messaging through the patient portal.

### Randomization

A randomization table was created prior to the start of recruitment to equally allocate 120 participants to 2 treatment groups. The first group received the intervention for 6 months and then returned to usual care (IV/UC) for 6 months. The second group received usual care for 6 months before enrolling in the intervention (UC/IV) for 6 months. Study staff not involved with recruitment created enrollment folders for each participant based upon the randomization table. Study staff responsible for recruitment were blinded to treatment group designation from study enrollment during baseline questionnaire administration. For participants randomized to receive the intervention during the first time period, the last baseline survey item asked if they would like to schedule a phone call with research staff to walk through using the connected glucose meter when they received it at home. Those interested were scheduled for a tutorial approximately 7 days later, after confirmed delivery of the intervention start-up package containing the connected glucose meter and testing materials. A similar tutorial request process occurred at the end of the 6-month survey for participants receiving the intervention during the second time period.

### Data Collection

At study enrollment, participants had an HbA_1c_ test drawn. Participants were scheduled to return at 3, 6, 9, and 12 months ±1 week post–study enrollment for HbA_1c_ testing. For participants who did not return for their scheduled 6-month (23/119, 19.3%) and 12-month (34/119, 28.6%) test, an HbA_1c_ recording from their closest clinical visit was extracted from the EHR if it was within 3 months of the scheduled lab testing date (49/57, 86% of total missing). For patients without an available HbA_1c_ in the EHR (8/57, 14% of total missing), change in HbA_1c_ was imputed with the mean of their treatment group in mixed-effects modeling analyses.

Participants completed paper questionnaires at baseline, 6 months (prior to treatment crossover), and 12 months (study completion). Participants were administered questionnaires at the clinic and could finish them at home and mail them back, if necessary. Data from the questionnaires were manually entered by study staff using REDCap data capture tools [11]. Data on engagement with intervention, including number of SMBG recordings, number of CDE contacts, and number of CDE coaching sessions were collected by Livongo and securely transferred to study staff for manual entry into the REDCap project.

### Primary and Secondary Outcomes

Changes in HbA_1c_ during each time period were the primary outcomes of this study. HbA_1c_ change was evaluated by comparing the mean changes in HbA_1c_ while receiving the IV compared to HbA_1c_ change while receiving UC. This was done for both the first treatment period and the second treatment period. Overall impact of the intervention on the change in HbA_1c_ across both time periods was assessed in a mixed-effects model.

Diabetes treatment satisfaction was chosen as a secondary outcome because it is associated with positive diabetes outcomes, including HbA_1c_ [12]. To measure baseline satisfaction with diabetes treatment, the Diabetes Treatment Satisfaction Questionnaire (DTSQ) was completed. The DTSQ is an 8-item measure with responses ranging from very satisfied to very dissatisfied for a total scale score range of 0 to 36 [13]. To evaluate change in satisfaction attributable to the intervention, the Diabetes Treatment Satisfaction Questionnaire Change (DTSQc) was included in the 6-month and 12-month questionnaires. The DTSQc is an 8-item measure that asks the extent to which participants experienced change in satisfaction over the course of the previous 6 months with responses ranging from much less satisfied now (−3) to much more satisfied now (+3) [14].

### Sample Size Estimation

The primary outcome of this study was change in HbA_1c_. We anticipated the distribution of change in HbA_1c_ would approximate a normal distribution, allowing for the use of a standard *t* test to examine differences in mean HbA_1c_ change between treatment groups during each time period. Based on previous interventions in this patient population [15,16], we assumed a 1.0% difference in mean HbA_1c_ change between treatment groups and a 1.5 SD in HbA_1c_ change for both groups, requiring 48 participants per group for 90% power and a type I error rate of .05. We assumed a 10% dropout, which required 53 participants per arm. A conservative approach targeted recruitment of 60 participants per treatment group. Sample size calculations were performed using the SAMPSI command in Stata software, version 13.1 (StataCorp).

### Analytic Plan

Bivariate comparisons of baseline characteristics between treatment groups were conducted to evaluate success of randomization. Baseline characteristics of the participants who failed to return for the 6-month and 12-month follow-up appointments were compared against those of participants who completed follow-up visits by using independent samples two-tailed *t* tests.

Primary outcome analyses involved independent samples two-tailed *t* tests to examine differences in HbA_1c_ change between treatment groups during the first and second time periods. Both intent-to-treat and completer analyses were conducted. Participants were considered completers if they returned for the 6-month and 12-month follow-up visits. To account for the crossover design and multiple time points of the study, a random intercept mixed-effects model with a restricted maximum likelihood estimator option of the mixed procedure in SAS software, version 9.4 (SAS Institute), was performed to examine variance between treatments by time with respect to subjects.

## Results

### Sample Characteristics

Study participants (n=119) had mean baseline HbA_1c_ of 10.1% (SD 1.4). Age at enrollment ranged from 23 to 84 years old with an average age of 56.7 years (SD 11.6). The study sample was 52.9% (63/119) women and 71.4% (85/119) white (Table 1). Both groups were similar in terms of demographic characteristics, insulin use, HbA_1c_, and treatment satisfaction.

**Table 1:** Study participants’ characteristics.

Characteristics		IV/UC^a^ (n=59)	UC/IV^b^ (n=60)	*P* value
Age (years), mean (SD)		56.1 (11.1)	57.4 (12.1)	.55
**Age (years), n (%)**				.56
	18-40	5 (8)	4 (7)	—^c^
	40-65	42 (71)	39 (65)	—
	65+	12 (20)	17 (28)	—
Gender (women), n (%)		34 (58)	29 (48)	.36
**Race, n (%)**				.65
	White	40 (68)	45 (75)	—
	Black	6 (10)	3 (5)	—
	Native/Alaskan American	1 (2)	0 (0)	—
	More than 1 race	7 (12)	6 (10)	—
	Not reported	5 (8)	6 (10)	—
**Ethnicity, n (%)**				.81
	Hispanic Latinx	11 (19)	9 (15)	—
	Not Hispanic Latinx	46 (78)	48 (80)	—
	Not reported	2 (3)	3 (5)	—
**Education, n (%)**				.80
	<High school grad	9 (15)	7 (12)	—
	High school grad	18 (31)	17 (28)	—
	Post–high school trade	6 (10)	5 (8)	—
	1-3 years college	14 (24)	16 (27)	—
	College grad	11 (19)	13 (22)	—
	Not reported	1 (2)	2 (3)	—
**Household income (US$), n (%)**				.78
	<20k	24 (41)	22 (37)	—
	20-50k	11 (19)	14 (23)	—
	50-100k	10 (17)	11 (18)	—
	>100k	11 (19)	7 (12)	—
	Not reported	3 (5)	6 (10)	—
**Internet access, n (%)**				.73
	No	9 (15)	11 (18)	—
	Yes	50 (85)	47 (78)	—
	Not reported	0 (0)	2 (3)	—
**Insulin use, n (%)**				.62
	No	7 (12)	9 (15)	—
	Yes	52 (88)	51 (85)	—
HbA_1c_^d^ %, mean (SD)		10.3 (1.4)	10.0 (1.4)	.21
Treatment satisfaction [14], mean (SD)		29.6 (5.3)	28.4 (5.2)	.24

^a^IV/UC: intervention for 6 months before usual care for 6 months.

^b^UC/IV: usual care for 6 months before intervention for 6 months.

^c^Not available.

^d^HbA_1c_: hemoglobin A_1c_.

### Study Retention

**Figure 2:**
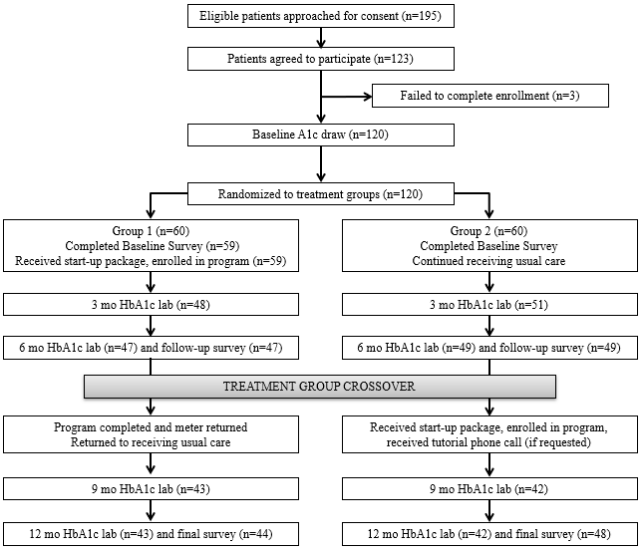
Participant CONSORT (Consolidated Standards of Reporting Trials) diagram. HbA_1c_: hemoglobin A_1c_.

Of the 119 study participants, 97 (81.5%) returned for the 6-month HbA_1c_ lab, and 92 (77.3%) completed the 6-month follow-up survey (Figure 2). After treatment crossover, 86 (72.3%) participants returned for the 12-month HbA_1c_ test, and 92 (77.3%) participants completed the 12-month follow-up survey. HbA_1c_ data from the nearest clinical appointment were extracted for 19 of the 22 (86%) participants who did not return for the 6-month HbA_1c_ lab and 30 of the 33 (91%) participants who did not return for the 12-month HbA_1c_ lab. HbA_1c_ values for the remaining participants with missing values at 6 months (n=3) and 12 months (n=3) were set to their group’s mean value so that the final analytic sample included follow-up HbA_1c_ data for all 119 participants at the 6-month and 12-month time points.

### Engagement With Intervention

Among participants randomized to receive the intervention first (IV/UC, n=60), 1 (2%) did not enroll in the intervention program, and 6 (10%) never used the intervention meter. Of the 60 participants randomized to receive the intervention in the second period (UC/IV), 11 (18%) did not complete the 6-month follow-up visit and subsequently failed to enroll in the intervention. Of those participants who enrolled in the intervention in the second period (n=49), 8 (16%) never used the meter.

Among all participants who used the intervention meter during either time period (n=94), the average number of SMBG recordings per participant over the 6-month intervention period was 220 (SD 165, range: 2-817). For these participants, 73 (78%) were contacted by an intervention CDE at least once in response to a high or low SMBG recording outside of range. Over the course of the entire study, 400 support contacts were attempted by intervention CDEs, with 295 (73.8%) successful contacts, defined as reaching the patient (phone call) or receiving a reply (text message). Of these, 183 (62.0%) were by phone, and 112 (38.0%) were by SMS text messaging. Among the 73 participants contacted in response to a flagged SMBG, 11 (15%) scheduled at least one follow-up coaching session with an intervention CDE. Among those who completed a coaching session with an intervention CDE, the average number of coaching sessions was 2.5 (SD 1.5) with a range from 1 to 5 total coaching sessions.

### Change in HbA_1c_

**Figure 3:**
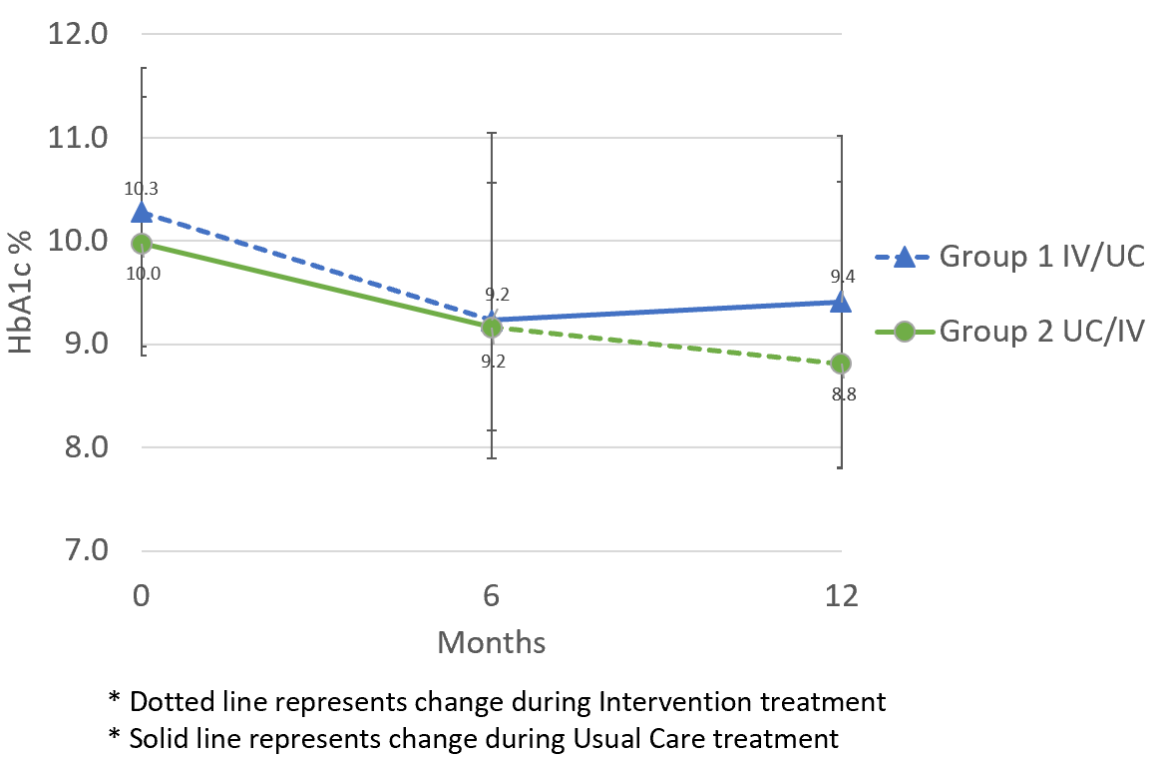
Mean HbA_1c_ % at 0, 6, and 12 months, by group*. HbA_1c_: hemoglobin A_1c_; IV: intervention; UC: usual care.

Similar rates of HbA_1c_ change were seen between both groups after 6 months (*t*_114_=1.06, *P*=.29), with the intervention improving mean HbA_1c_ by 1.1% (SD 1.5; *P*<.001) and usual care by 0.8% (SD 1.5; *P*<.001) (Table 2). After crossover, those returning to usual care (IV/UC) did not experience significant change in mean HbA_1c_ (*P*=.41), while those who began receiving the intervention (UC/IV, n=39) had additional improvement in mean HbA_1c_ by 0.4% (SD 1.0; *P*=.008) (Figure 3). The difference in mean HbA_1c_ change during the second time period between groups was not statistically significant in intent-to-treat analyses (*P*=.09) but was significant among the participants who completed the final study visit (*P*=.03) (Table 2).

**Table 2:** Change in HbA_1c_ percentage and diabetes treatment satisfaction, by group.

Outcome		IV/UC^a^		UC/IV^b^		*P* value
		n	Mean (SD)	n	Mean (SD)	
**Baseline**						
	HbA_1c_^c^ %	59	10.3 (1.4)	60	10.0 (1.4)	.25
	DTSQ^d^	56	29.6 (5.3)	59	28.4 (5.2)	.24
**6-month follow-up**						
	∆ HbA_1c_ % from baseline (ITT^e^)	56	−1.1 (1.5)	60	−0.8 (1.5)	.29
	∆ HbA_1c_ % from baseline (completer)	47	−1.1 (1.5)	49	−0.7 (1.3)	.14
	DTSQc^f^	42	+12.9 (5.5)	46	+10.7	.09
**12-month follow-up**						
	∆ HbA_1c_ % from 6-month (ITT)	56	+0.2 (1.7)	60	−0.4 (1.5)	.07
	∆ HbA_1c_ % from 6-month (completer)	41	+0.3 (1.7)	39	−0.4 (1.0)	.03
	DTSQc	40	+11.5 (6.8)	42	+13.4 (5.8)	.15

^a^IV/UC: intervention for 6 months before usual care for 6 months.

^b^UC/IV: usual care for 6 months before intervention for 6 months.

^c^HbA_1c_: hemoglobin A_1c_.

^d^DTSQ: Diabetes Treatment Satisfaction Questionnaire.

^e^ITT: intent-to-treat.

^f^DTSQc: Diabetes Treatment Satisfaction Questionnaire Change.

The mixed-effects model (Table 3) showed a nonsignificant difference in HbA_1c_ improvement of 0.4% between the intervention and usual care treatment conditions (*P*=.06). The model also showed significant effects of baseline HbA_1c_ (*P*=.03) and time period (*P*<.001). Participants with higher baseline HbA_1c_ saw greater HbA_1c_ improvement across the whole study, and there was greater HbA_1c_ improvement in the first period compared to the second period.

**Table 3:** Results of crossover (mixed-effects model) analysis of HbA_1c_ change.

Variable	HbA_1c_^a^ % change estimate	SD	*P* value
Baseline HbA_1c_	−0.15	0.07	.03
Treatment (IV^b^ vs UC^c^)	−0.37	0.19	.06
Time period (1 vs 2)	−0.84	0.20	<.001
Treatment × period	0.29	0.39	.46

^a^HbA_1c_: hemoglobin A_1c_.

^b^IV: intervention.

^c^UC: usual care.

### Change in Diabetes Treatment Satisfaction

Among participants completing the 6-month questionnaire (n=96), those receiving the intervention reported a mean improvement in treatment satisfaction of +12.9 (SD 5.6) compared to +10.7 (SD 6.6) with usual care (*P*=.09). Among those completing the final questionnaire (n=82), those who returned to usual care in the second time period (IV/UC) reported an improved mean treatment satisfaction change score of +11.5 (SD 6.8) compared to +13.4 (SD 4.5) among participants who received the intervention in the second time period (UC/IV, *P*=.15).

## Discussion

### Principal Results

In this 12-month randomized crossover trial, we found that patients enrolled in a diabetes remote monitoring program experienced improvements in HbA_1c_ and treatment satisfaction similar to usual care at a specialty diabetes center. Our mixed-effects model assessing HbA_1c_ change over both 6-month time periods estimated that HbA_1c_ improvement produced by the intervention was approximately 0.4% greater than that produced by usual care, though not reaching statistical significance (*P*=.06). At the same time, we did not observe differences in treatment satisfaction between the program and usual care. Together, these findings provide additional evidence regarding the expected outcomes of a commercial remote monitoring program, which may be useful for health organizations and insurers to consider in making decisions for patient self-management support.

In the first 6 months, patients experienced improvement in HbA_1c_, including those receiving usual care, who exhibited improvement in mean HbA_1c_ by −0.8%. This is a common finding in comparable trials involving patients with uncontrolled diabetes and may result from multiple factors. First, improvement through usual care could be due to the Hawthorne effect [17]. Participants received additional attention and engaged frequently with research staff, they were called and reminded to return quarterly for HbA_1c_ testing, and they knew they would receive the anticipated commercial intervention after 6 months. Second, patients received specialized care through the DCOE endocrinologists and may represent more intensive blood glucose management than typically experienced through the primary care setting. This and potential “spillover” effects may have additionally narrowed differences observed between treatment conditions. Finally, “regression to the mean” may have contributed to improvements in all patients by recruiting only those with higher baseline HbA_1c_ levels to the study.

### Comparison With Prior Work

As in other studies, patients who missed follow-up visits for data collection had higher baseline HbA_1c_ levels. For these individuals, it is not clear that commercial programs adequately address the barriers to complex diabetes self-management behaviors and social determinants of health, particularly with remote CDE support. Program CDEs may not develop the same relationships with patients as health care team members or recognize cultural, regional, or other psychosocial issues that may influence glycemia. Unfortunately, in many health care settings these patients still tend to have high no-show rates for appointments, worse diabetes-related health outcomes, lower rates of SMBG testing, and greater medication nonadherence [18-20].

Similar interventions involving SMBG and targeting patients with poorly controlled diabetes have demonstrated improvement in health outcomes for this increasingly prevalent and costly patient population [15,16,21-27]. Unique to this intervention was the in-the-moment, virtual support provided in response to abnormal SMBG levels uploaded automatically by connected glucose meters. By contacting patients immediately after their blood glucose tests high or low, CDEs could offer timely support when patients may need it most (eg, immediate hypoglycemia treatment). The CDE could also take advantage of “teachable moments” to provide diabetes education and self-management training when there is greater attention [28]. During these unplanned opportunities, patients can gain a better understanding of why their blood glucose is outside of range and learn how best to prevent it from happening again in the future.

While timely CDE outreach may be useful for some patients, it could also prompt stress in those who may not want to be contacted when SMBG levels are out of range. To address this concern, participants could adjust the SMBG levels that would trigger CDE contact; however, no participants requested to do this during the study. This may be secondary to following a “default” (status quo bias) [29] or may be due to a lack of technological knowledge on how to fully operate the meter. As a result, it remains possible that individuals will avoid self-testing if they suspect their levels are more extreme to avoid CDE involvement, especially if they exhibit more risk-seeking behavior [30]. If true, it suggests that for future implementation, this option should be emphasized upon initial training or reassessed over time.

Similarly, we found that only a small proportion of participants scheduled an individual coaching session with a program CDE. Routine scheduled coaching sessions for all participants may further enhance delivery of diabetes self-management education and training in this population. Additionally, CDEs could contact and counsel patients who have not recorded an SMBG level over an extended period. Besides the CDEs, the program could encourage greater involvement of a patient’s care team and support system, including informal caregivers such as family members. Providing caregivers with electronic access to a patient’s SMBG recordings and tools to assist in disease management may improve the quality of support they provide and reduce their own caregiver burden. We did not investigate the effects of this intervention on caregiver support and burden, but this should be considered in a future study.

### Strengths

There were several strengths in this study. We collected both physiological (HbA_1c_) and patient-reported (diabetes treatment satisfaction) outcomes. Prior study of the program only included detection of glucose levels outside of range and excluded treatment satisfaction [8]. Additionally, the randomized controlled crossover study design allowed for both between- and within-group comparisons. This provided a more comprehensive evaluation by time period, treatment, and sequence of treatment received. Finally, we built an application programming interface to allow the transfer of SMBG and CDE/patient interactions from the Livongo cloud-based system to the clinic’s EHR. This allowed for the intervention data to be accessible to the patients’ care teams between clinic appointments.

### Limitations

There are several limitations of this study to consider. The intervention time period was relatively short (6 months) for a group of patients with poorly controlled diabetes receiving care at a specialty diabetes center. The limited exposure to the intervention did not allow for evaluation of a sustained intervention effect. In addition, as only patients receiving the intervention had SMBG recordings regularly uploaded, we did not compare frequency of blood glucose testing during intervention compared to usual care. More research is needed with longer durations of intervention treatment, as most studies are 12 months or less [6], and in other patient populations, as this study only focused on patients with poorly controlled diabetes and did not collect data on duration of diabetes at time of enrollment. Second, data analyzed are from 2015 to 2016, and the intervention program has made several adaptations since study completion. Livongo has partnered with several companies recently, including Dexcom and their continuous glucose monitoring (CGM) devices and Fitbit with their physical activity trackers. Furthermore, Livongo recently merged with Teladoc Health, a leading telemedicine provider. Further study of Livongo’s effect after incorporating CGM devices, wearable devices with more telehealth human coaching activities and advanced decision support, is needed. This is especially important considering a very limited number of participants in this study took advantage of a scheduled coaching session.

As well, while accessibility to virtual diabetes care support programs like Livongo has increased recently, many patients may continue to face barriers accessing or affording such support. These access to care challenges limit the generalizability of the study to only patients with access to such programs. Additionally, this study did not target provider behavior. SMBG data was uploaded to the EHR daily, but optimizing the use of these data by the usual care team was not part of the intervention. In regard to retention, several participants failed to return for their 6-month visit (28%), with those in the UC/IV group never receiving the intervention during the second study time period. Lastly, there may have been carryover of treatment effects for participants who received the intervention first (IV/UC), especially considering the absence of a washout period in the study design.

### Conclusions

We found that patients with poorly controlled diabetes enrolled in the commercial remote diabetes monitoring program experienced improvements in HbA_1c_ similar to when they received usual care at a specialized diabetes center. Improved treatment satisfaction was also reported by both groups throughout the study. Further development targeting patient engagement in the program and access to CDEs for diabetes support could result in greater program impact, especially for patients with limited access to specialized diabetes care. Future interventions involving diabetes care monitoring programs and connected technologies should consider including a structured coaching component, proactively involving caregivers and family members of patients, and investing in additional efforts to engage patients who are more likely to miss scheduled study activities and appointments. Better integration of diabetes remote monitoring programs into routine clinical care must be prioritized. This is necessary in order to achieve the full potential benefit from similar interventions in the future. In addition, cost-effectiveness needs to be investigated. This will be critical in justifying the expense required to provide in-the-moment support offered by the intervention.

## Appendix

Multimedia Appendix 1 CONSORT EHEALTH checklist.

## Abbreviations

AADE: American Association of Diabetes Educators
CDE: Certified Diabetes Educator
CGM: continuous glucose monitoring
DCOE: Diabetes Center of Excellence
DTSQ: Diabetes Treatment Satisfaction Questionnaire
DTSQc: Diabetes Treatment Satisfaction Questionnaire Change
EHR: electronic health record
HbA_1c_: hemoglobin A_1c_
IV: intervention
UC: usual care
SMBG: self-monitoring of blood glucose
T2D: type 2 diabetes
